# The Thymus Is a Common Target Organ in Infectious Diseases

**DOI:** 10.1371/journal.ppat.0020062

**Published:** 2006-06-30

**Authors:** Wilson Savino

**Affiliations:** University of British Columbia, Canada

## Abstract

Infectious disease immunology has largely focused on the effector immune response, changes in the blood and peripheral lymphoid organs of infected individuals, and vaccine development. Studies of the thymus in infected individuals have been neglected, although this is progressively changing. The thymus is a primary lymphoid organ, able to generate mature T cells that eventually colonize secondary lymphoid organs, and is therefore essential for peripheral T cell renewal. Recent data show that normal thymocyte development and export can be altered as a result of an infectious disease. One common feature is the severe atrophy of the infected organ, mainly due to the apoptosis-related depletion of immature CD4^+^CD8^+^ thymocytes. Additionally, thymocyte proliferation is frequently diminished. The microenvironmental compartment of the thymus is also affected, particularly in acute infectious diseases, with a densification of the epithelial network and an increase in the deposition of extracellular matrix. In the murine model of Chagas disease, intrathymic chemokine production is also enhanced, and thymocytes from Trypanosoma cruzi-infected mice exhibit greater numbers of cell migration-related receptors for chemokines and extracellular matrix, as well as increased migratory responses to the corresponding ligands. This profile is correlated with the appearance of potentially autoreactive thymus-derived immature CD4^+^CD8^+^ T cells in peripheral organs of infected animals. A variety of infectious agents—including viruses, protozoa, and fungi—invade the thymus, raising the hypothesis of the generation of central immunological tolerance for at least some of the infectious agent-derived antigens. It seems clear that the thymus is targeted in a variety of infections, and that such targeting may have consequences on the behavior of peripheral T lymphocytes. In this context, thymus-centered immunotherapeutic approaches potentially represent a new tool for the treatment of severe infectious diseases.

## Introduction

### 

Immunology of infectious diseases has focused mainly on the effector immune response, changes in the blood and peripheral lymphoid organs of infected individuals, and vaccine development. In comparison, studies on the thymus under the biological pressure of an infectious agent have been few, although the large amount of data recently published on the thymus of HIV-bearing patients promises to improve this deficiency.

In the present review we will discuss a number of findings related to the lymphoid as well as the microenvironmental compartments of the thymus in the infectious disease state (including the thymic invasion by some infectious agents), as well as the phenotypic and functional changes seen with each of these intrathymic cellular compartments, comprising proliferation, death, secretion, migration, and differentiation. Selected examples of viral and parasitic infectious diseases will be discussed in more detail. Nevertheless, before compiling and discussing these data, it is worthwhile to provide a general background on the structure of the thymic microenvironment and its role in intrathymic T cell differentiation.

#### The thymic microenvironment and T cell differentiation.

The thymus is a primary lymphoid organ in which bone marrow-derived T cell precursors undergo differentiation, ultimately leading to migration of positively selected thymocytes to the T cell-dependent areas of peripheral lymphoid organs. This process involves sequential expression of various proteins and rearrangements of T cell receptor (TCR) genes.

Along with differentiation, the most immature thymocytes express neither the TCR complex nor the CD4 or CD8 accessory molecules; they are called double-negative (CD4^−^CD8^−^) cells and represent 5% of total thymocytes. Maturation progresses with the acquisition of CD4 and CD8 markers, generating CD4^+^CD8^+^ double-positive cells, which constitute 80% of the whole population. At this stage, TCR genes are completely rearranged, and productive rearrangements yield the membrane expression of TCRs (complexed with CD3) at low densities (TCR^low^). Thymocytes that do not undergo a productive TCR gene rearrangement die by apoptosis, whereas those expressing productive TCRs interact with peptides presented by molecules of the major histocompatibility complex (MHC), expressed on microenvironmental cells. This interaction determines the positive and negative selection events that are crucial for normal thymocyte differentiation. Positive selection allows the differentiation step through which immature, short-lived, CD4^+^CD8^+^ thymocytes escape from programmed cell death and become mature, long-lived, CD4^+^ or CD8^+^ single-positive cells. This is a highly stringent process, sparing only a small proportion of the CD4^+^CD8^+^ population. Positive selection also coincides with lineage commitment: the decision to become a CD4^+^ or CD8^+^ single-positive thymocyte, as a function of the class of MHC molecule with which the TCR can interact. Intrathymic negative selection is the screen that allows the establishment self-tolerance in the T cell repertoire, promoting apoptosis-mediated deletion of most T cells that might potentially react to self proteins.

**Figure 1:**
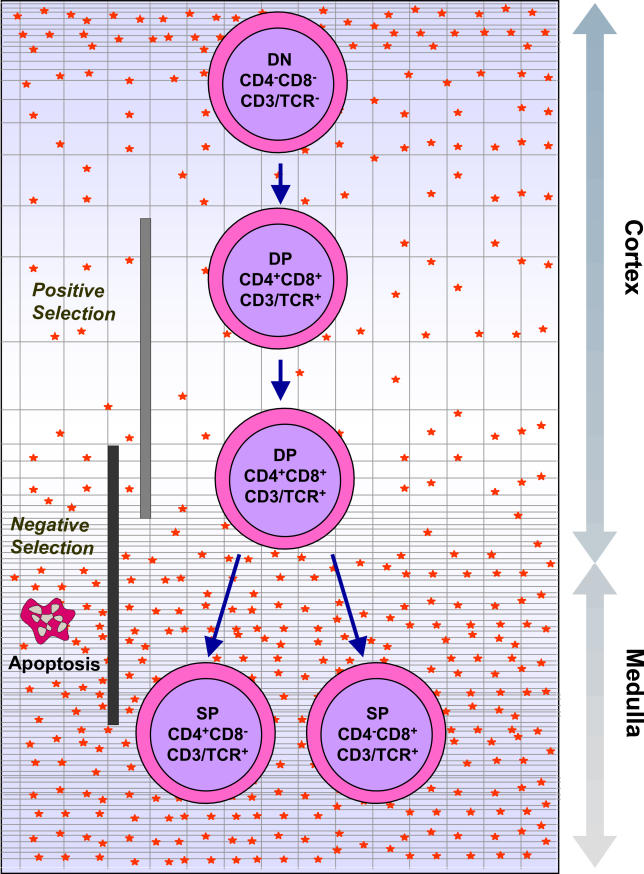
The Normal Process of Intrathymic T Cell Differentiation This diagram shows that most immature thymocytes localized in the subcapsular cortical region of the thymic lobules do not express CD4 or CD8 accessory molecules, nor the CD3/TCR complex, and are known as double-negative (DN, for CD4 and CD8) cells. As they progress in differentiation, they begin to express on their cell membranes the TCR/CD3 complex as well as CD4 and CD8, becoming double-positive (DP) thymocytes, which occupy most of the cortical region. These cells are then submitted to the processes of positive and negative selection, as a consequence of the interaction with the thymic microenvironment (gray network) through MHC-TCR interactions. Those cells undergoing negative selective die by apoptosis, whereas the small percentages of positively selected thymocytes progress in their differentiation, moving toward the medulla and becoming single-positive cells (SP) for either CD4 or CD8, both expressing high densities of CD3/TCR complex. These mature thymocytes can be exported from the thymus into the peripheral lymphoid organs. Finally, this overall process of thymocyte differentiation occurs in the context of the three-dimensional thymic microenvironment (gray network) through membrane interactions as well as soluble products (represented by red stars) released by microenvironmental cells. Modified from [9].

Positively selected thymocytes that progress to the mature TCR^high^CD4^+^CD8^−^ or TCR^high^CD4^−^CD8^+^ single-positive stage constitute 15% of thymocytes that ultimately leave the organ to form the large majority of the peripheral T cell repertoire [1–4]. Figure 1 is a simplified view of the sequential steps of thymocyte differentiation within the context of the nonlymphoid compartment, the thymic microenvironment.

It is noteworthy that a small minority of potentially self-reactive thymocytes achieves the CD4 or CD8 single-positive stage and are released from the organ. Accordingly, along with differentiation into CD4^+^ single positive cells, some elements do not acquire the functional feature of typical helper cells (that is, cells able to trigger and/or enhance an immune response in the periphery), but rather differentiate into “regulatory” cells (most of them bearing the phenotype CD4^+^CD25^+^FoxP3^+^), which actually block a given immune response. Recent data show that defects in such regulatory CD4^+^ T cells may be related to the occurrence of autoimmune events (reviewed in [5–7]).

**Figure 2:**
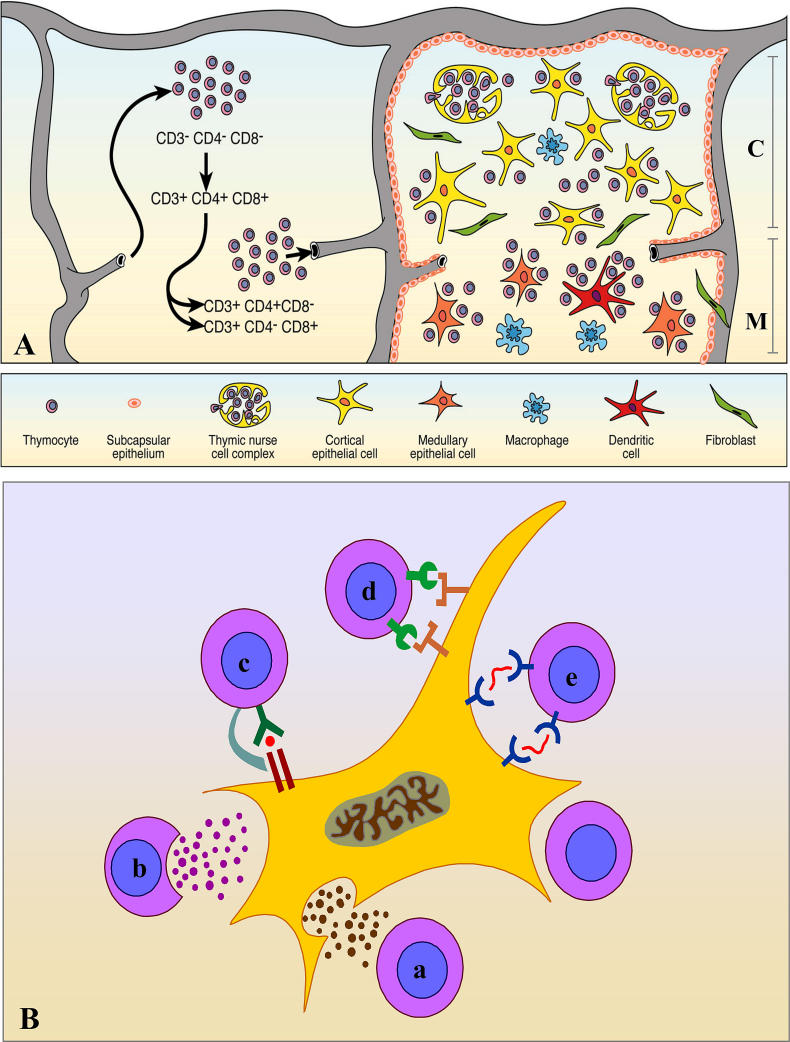
The Thymic Microenvironment and Its Role in Thymopoiesis (A) A simplified model of thymocyte migration includes two compartments. On the left is depicted the entrance site of precursor cells into the thymus through blood vessels. Having entered the thymus, thymocytes migrate during differentiation to ultimately leave the organ, bearing the mature phenotypes of CD4^+^CD8^−^ or CD4^−^CD8^+^ cells. The right side of the image is a schematic representation of a thymic lobule, showing thymocytes intermingled with a heterogeneous cellular network, the thymic microenvironment, composed of epithelial cells (yellow and orange), dendritic cells (red), macrophages (blue), and fibroblasts (green). The epithelial tissue shows morphologic heterogeneity that can be seen in subseptal/subcapsular, cortical, and medullary regions. In the cortex, we note a particular lymphoepithelial complex, the TNC. (B) A number of molecular interactions take place between developing thymocytes and thymic epithelial cells. Whereas a and b correspond to interactions mediated by soluble secretory molecules produced by epithelial cells (a) or lymphocytes (b), the interaction shown in c involves a given peptide (red dot) being presented by MHC (expressed by the epithelial cell) to the TCR and corresponding accessory molecule in the thymocyte membrane. The interaction shown by (d) involves adhesion molecules and the respective membrane counter-receptors, and (e) depicts an interaction mediated by ECM ligand and receptor. Modified from [3,8].

Thymocyte differentiation occurs as cells migrate within the thymic lobules: TCR^−^CD4^−^CD8^−^ and TCR^+^CD4^+^CD8^+^ are cortically located, whereas mature TCR^+^CD4^+^CD8^−^ and TCR^+^CD4^−^CD8^+^ cells are found in the medulla (Figure 2). As this journey proceeds, thymocytes interact with various components of the thymic microenvironment, a three-dimensional network formed of thymic epithelial cells (TECs), macrophages, dendritic cells, fibroblasts and extracellular matrix (ECM) components (Figure 2A).

In addition to the key interaction involving the TCR/peptide-MHC, in the context of CD8 or CD4 molecules the thymic microenvironment influences thymocyte maturation via adhesion molecules and ECM; these interactions are relevant for thymocyte migration [8,9]. Moreover, microenvironmental cells modulate thymocyte differentiation by soluble polypeptides, comprising (a) typical cytokines, such as interleukin (IL)-1, IL-3, IL-6, IL-7, IL-8 and stem cell factor; (b) chemokines, including CXCL12, which preferentially attracts immature CD4^−^CD8^−^ and CD4^+^CD8^+^ thymocytes, and CCL21, that exerts chemoattraction for mature single positive thymocytes [10–12]; and (c) thymic hormones such as thymulin, thymopoietin, and thymosin-α1, that can also act on the general process of thymocyte maturation [3,13]. Interestingly, not only the thymic epithelium affects thymocyte behavior, but thymocytes modulate some thymic epithelial functions, as exemplified by the role of interferon-γ in the expression of MHC molecules, extracellular ligands, and receptors [14,15]. The various TEC/thymocyte interactions are summarized in Figure 2B, and Table 1 summarizes accession numbers of peptide and DNA sequence databases of selected human and mouse proteins cited throughout this manuscript.

**Table 1:**
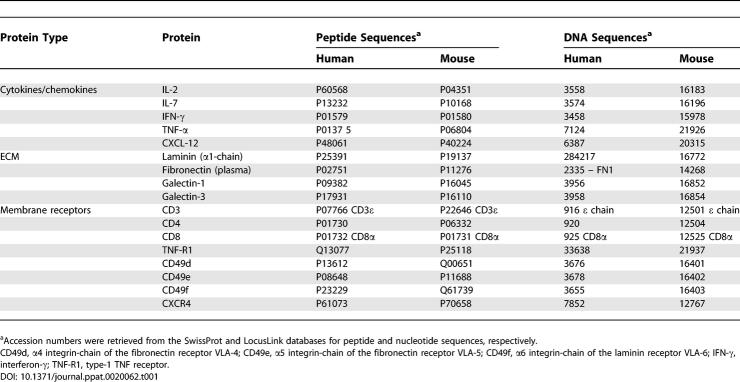
Protein and Gene Accession Numbers of Selected Proteins

The thymic epithelial network is the major component of the thymic microenvironment, and it is responsible for positive selection of thymocytes. It is a morphologically and phenotypically heterogeneous tissue, and cells in different locations within the thymic lobules may be related to specific steps in T cell maturation [2]. One cortically located lymphoepithelial complex, the thymic nurse cell (TNC), has been isolated in vitro. TNCs are lymphoepithelial multicellular structures formed by one TEC (which in mice can harbor 20–200 thymocytes), and are located in the cortical region of thymic lobules. Most intra-TNC lymphocytes bear the CD4^+^CD8^+^ double-positive phenotype, although immature double-negative as well as mature single-positive cells can be found. TNCs may create special microenvironmental conditions for thymocyte differentiation and/or proliferation, and within this complex distinct interactions occur, comprising those mediated by soluble products, ECM, and MHC/TCR [3]. Once settled in culture, TNCs spontaneously release thymocytes, and TNC-derived epithelial cells can reconstitute lymphoepithelial complexes after being cocultured with immature thymocytes [16]. Thus, TNCs constitute an in vitro model of thymocyte migration within the TEC context.

The thymocyte differentiation process and the thymic microenvironment compartment can be regarded as potential targets for direct or indirect effects of a given infectious agent.

#### Thymic atrophy is a common feature in infectious diseases.

**Figure 3:**
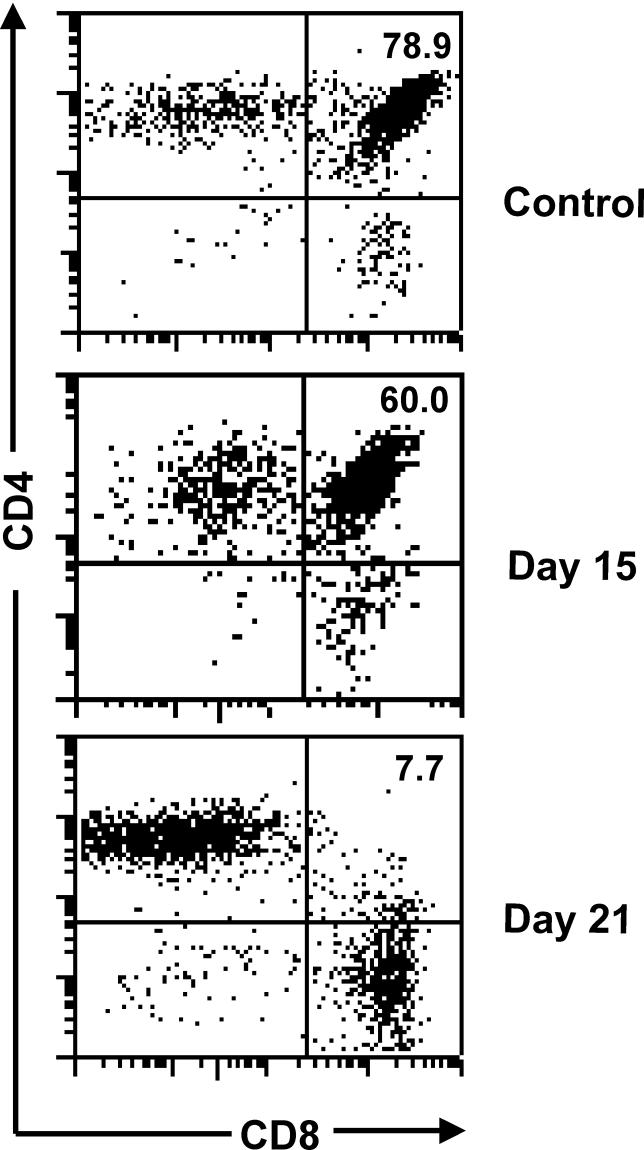
Progressive Thymic Atrophy in Mice Acutely Infected by T. cruzi A typical CD4/CD8-defined cytofluorometric profile of normal thymocytes compared to that with T. cruzi infection. As infection progresses, we can see a progressive loss of CD4^+^CD8^+^ cells. Percentage values of the CD4^+^CD8^+^ subset are shown within the quadrants. The days correspond to the time of infection, with an inoculum of 10^5^ parasites per animal. The peak of parasitemia coincides with the peak of thymocyte depletion. Modified from [43].

A common feature seen in a variety of acute infections is severe atrophy of the thymus, largely reflecting intense lymphocyte depletion, particularly of cortical thymocytes bearing the phenotype CD4^+^CD8^+^ (Figure 3 and Table 2). This depletion actually corresponds to massive cortical thymocyte apoptosis, as it has been shown in a variety of infections, including viral diseases such as AIDS, simian immunodeficiency syndrome, and rabies; experimental bacterial infections such as turalemia and listeriosis; diseases caused by parasites including *T. cruzi, Plasmodium chaubi, Schistosoma mansoni,* and *Trichinella spiralis;* and fungal infections, exemplified by experimental infections with Paracoccidioides brasiliensis and *Histoplasma capsulatum* [17–36]. In some cases, thymocyte loss is so great that the cortical region of thymic lobules virtually disappears as a consequence of the severe CD4^+^CD8^+^ thymocyte depletion.

**Table 2:**
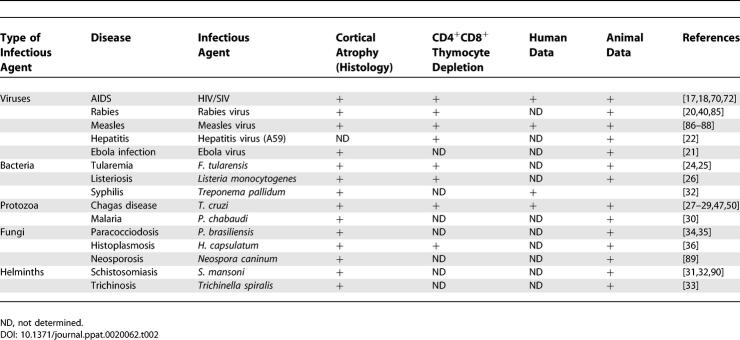
Thymic Atrophy in Human and Experimental Infectious Diseases

The precise mechanisms responsible for the thymic atrophy seen in acute infections are not completely elucidated, and may vary in distinct diseases. One major pathway is related to the rise in glucocorticoid hormone levels in the blood, a classical effect comprised within the organism's stress response to the infection. It is well known that such steroids can trigger apoptosis in thymocytes, acting via a specific receptor, a ligand-activated transcription factor [37]. CD4^+^CD8^+^ thymocytes are particularly sensitive to glucocorticoids, with the activation of caspase-3, −8, and −9 [38], whereas mature single-positive thymocytes are much more resistant, through a mechanism dependent on CD28 signaling [39].

Thymocyte depletion in rabies virus-infected mice [40] can be prevented by adrenalectomy prior to infection, clearly indicating that in this case, cell death is related to increased serum glucocorticoid [41]. Ablation of the adrenal glands also prevents the thymocyte depletion seen in the experimental infection by the bacterium Francisella tularensis [24]. Nevertheless, in this case, tumor necrosis factor (TNF)-α also seems to be involved, since thymic atrophy was not seen in TNR receptor-deficient mice infected with F. tularensis [25]. Nevertheless, in the murine model of experimental Chagas disease, despite the high levels of corticosterone seen in acutely and chronically infected animals [28,42], adrenalectomy did not prevent T. cruzi-induced cortical thymocyte depletion.

The relative role of glucocorticoids upon intrathymic cell death seen in several acute infections deserves to be revisited. As we summarized above, currently available data are based on the rise of serum glucocorticoid hormone levels, as well as on adrenalectomy experiments. These indicators do not take into account the intrathymic production of functional glucocorticoids by thymic epithelial cells (reviewed in [37,43,44]). Accordingly, it is completely unknown whether the intrathymic levels of glucocorticoids vary in infected individuals, and if so, what the local consequences are in terms of the general process of thymocyte differentiation.

As already exemplified by experimental tularemia, other death-related molecular pathways (such as the one triggered by TNF) may be involved in the generation of thymic atrophy seen in infectious diseases. Unfortunately, as regards experimental T. cruzi infection, the putative role of TNF-α in thymocyte depletion has not been investigated so far. This topic should be studied, in view of the data showing the enhanced intrathymic contents of this death-related cytokine [45], and our recent findings that TNF-α is actually involved in the CD8^+^ T cell apoptosis seen in mesenteric lymph nodes from T. cruzi-infected mice [46].

Studies performed in Fas-deficient *gld/gld* as well as in perforin knockout mice revealed a significant thymic atrophy upon T. cruzi infection, thus discarding the involvement of interactions mediated by Fas/Fas-L or perforin in triggering cell death within the thymus [47]. By contrast, two distinct mechanisms have been implicated in the thymic atrophy occurring in experimental Chagas disease. A nonvirulent strain of the parasite did not induce thymic atrophy or thymocyte depletion [48], suggesting that parasite-derived factors could be involved. This result is in keeping with the data showing that the specific treatment of the disease with benznidazole prevents intrathymic CD4^+^CD8^+^ cell depletion [49]. Accordingly, Mucci and coworkers [50] observed that apoptosis seen in thymic nurse cell complexes from infected mice could be due to the parasite-derived enzyme trans-sialidase. In a second study, it was showed that thymocytes from infected mice were particularly sensitive to the proapoptotic action of extracellular ATP, acting through the P2X_7_ purinergic receptor [51]. Moreover, we recently noticed that thymocyte depletion associated with T. cruzi infection was not seen in galectin-3 knockout mice (unpublished data), thus suggesting a role for this molecule in the infection-induced thymocyte loss, in addition to its de-adhesion role [52]. Conjointly, these data indicate that depletion of thymic lymphocytes may result from multiple interactions involving both endogenous and infectious agent-derived moieties. As further discussed below, it is noteworthy that several infectious agents can reach the thymic parenchyma, as it has been shown for some viruses, protozoa, and fungi [27,32,34,53].

Very few studies have been done to address the question of the fate of dead thymocytes seen in infectious diseases. Nevertheless, most likely they are phagocytosed by intrathymic macrophages, as it has been demonstrated following infection of macaques with simian immunodeficiency virus (SIV) [18].

Lastly, it is worthwhile to mention that coinfection may have an impact on thymocyte apoptosis. Although literature on this issue is very scarce, it has been shown that coinfection of mice with hepatitis virus type 3 and T. cruzi yielded a higher degree of apoptosis (ascertained by in situ TUNEL labeling) than that of each infection alone [48]. Considering the growing medical importance of coinfection in AIDS, this point certainly deserves further investigation.

#### Intrathymic cell proliferation and cytokine production in infectious diseases.

In addition to intrathymic apoptosis, which takes place in a variety of experimental and human infectious diseases, mitogenic responses of thymocytes can be altered. We found a significant decrease in both concanavalin A- and anti-CD3-driven proliferative responses in thymocytes from T. cruzi-infected mice compared to controls. This decrease was paralleled by a decrease in IL-2 production [54]. At the same time, we observed an increase in IL-10 as well as IFN-γ secretion by thymocytes from infected animals that originated from the decreased IL-2 and consequent diminished proliferative response: the in vitro treatment of cultured thymocytes from T. cruzi-infected animals with blocking antibodies to IL-10 and IFN-γ did restore IL-2 production and thymocyte proliferation induced by mitogens [54]. In a second vein, the ex vivo increase in the production of IL-4, IL-5, and IL-6 could be at least partially involved in the appearance of cytotoxic activity seen in thymuses from infected mice [54].

Experiments performed in SCID-hu mice (mouse chimeras bearing human T cells derived from transplantation of human thymic fragments and liver tissue under the renal capsule) revealed an increase of IL-6 and IFN-γ mRNA in thymocytes from HIV-infected mice, where IL-2 mRNA was decreased as ascertained by conventional RT-PCR [55]. This study also showed that antiretroviral therapy tended to increase the levels of cytokine mRNAs and to restore the proportions of the various CD4/CD8-defined thymocyte subpopulations.

#### The thymic microenvironment in infectious diseases.

In addition to the changes seen in thymic lymphocytes in various infectious diseases, the microenvironmental compartment of the organ can be affected. In experimental T. cruzi infection, for example, we noticed changes in TEC phenotypes: some cortical TECs expressed cytoskeletal markers normally restricted to the medullary epithelium. Simultaneously, epithelial cells in the medulla expressed the cytokeratin pair 8/18, which is restricted to cortical TECs in normal conditions [27]. It is noteworthy that such changes are not specific of the infection by *T. cruzi,* since they were also found in mice infected with Schistosoma mansoni [32].

In some viral infections, such as those caused by HIV and measles virus, the thymic epithelium is also severely damaged, with changes in phenotype and induction of apoptosis in adjacent thymocytes [17,56]. Studies performed with in vitro infection of human TECs by measles virus revealed that, in addition to inducing apoptosis in thymocytes, the virus arrests cell growth and induces terminal differentiation of the thymic epithelium [56].

In keeping with the densification of the TEC network, seen in vivo following some acute infections, including experimental Chagas disease, the MHC class II meshwork seen in the thymic microenvironment is also denser than the profiles seen in control thymuses [27]. Although solid data are scarce, this pattern is likely a general one in acute infections that generate atrophy of the thymic lobules, and thus it should be placed in the context of interactions with adjacent lymphocytes. It is conceivable that a denser MHC network results in an altered presentation of endogenous peptides to developing thymocytes, yielding alterations in the genesis of the intrathymic T cell repertoire. This issue will be further discussed below.

With respect to soluble products, we observed in *T. cruzi-*infected mice a transient decrease in the serum levels of the thymic hormone thymulin [27], known to be restrictedly produced by TECs [3]. In human HIV infection a consistent and long-term diminution of thymulin secretion has also been documented, in terms of both serum levels and intrathymic contents of the hormone [17,57,58].

Concerning microenvironmentally derived cytokines, studies using experimental HIV infection in humanized immunodeficient SCID-hu mice (pretransplanted with human thymic fragments) revealed an increase in IL-6 mRNA in the microenvironmental compartment of the thymus, although the levels of CXCL12 mRNA were not significantly altered [55].

An interesting feature is that interaction of thymocytes with thymic epithelial cells seems to be required for HIV replication in humans [59], leading to the secretion of various cytokines by the thymic epithelium, including IL-7, a major soluble factor in intrathymic T cell differentiation. In fact, the same research group showed more recently that TEC-derived IL-7 is able to up-regulate the expression of the CXC12 receptor CXCR4 by mature CD4^+^ single-positive thymocytes, which favors HIV replication in these cells [60].

Regarding TEC-derived chemokines, in a recent study we noticed in T. cruzi-acutely infected mice an increase in the intrathymic contents of CXCL12 concomitant with an increase in the membrane density of CXCR4 in the corresponding thymocytes [12,61]. Unfortunately, we have not measured IL-7 production by TECs of infected animals or cultures; this study should be instructive in better understanding the mechanisms leading to enhancement of CXCR4 expression.

**Figure 4:**
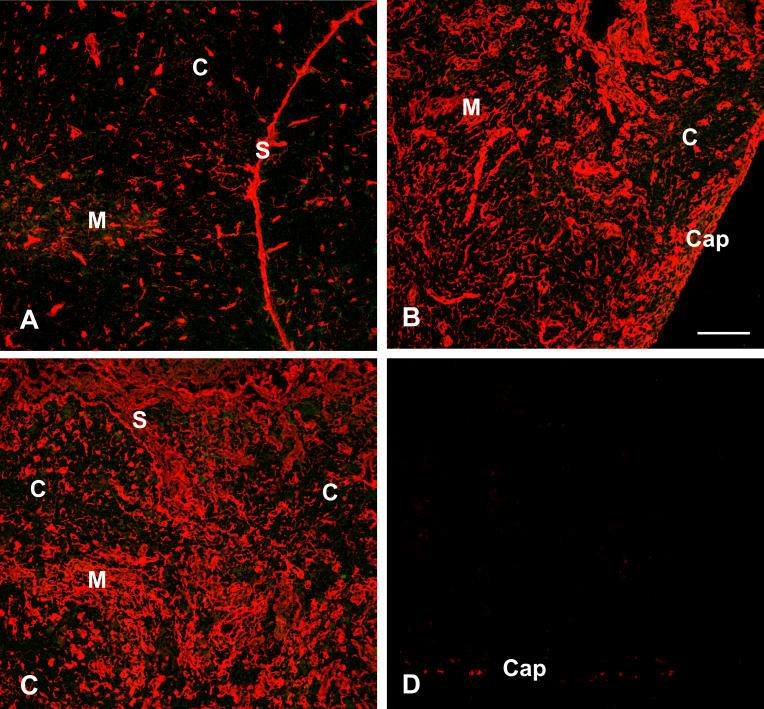
Increase in Intrathymic ECM Following Acute T. cruzi Infection in Mice Cryostat sections of thymuses from normal (A) or T. cruzi-infected mice (B, C, and D) were immunostained with anti-fibronectin immune serum (A, B, and C) or unrelated antibody (D). In control thymus (A), the typical fibronectin-containing network is seen, being more prominent in the medullary region of the thymic lobule (M), as compared to the cortex (C). This pattern is dramatically changed in the atrophic thymuses from T. cruzi-infected animals (B and C), in which the fibronectin network is much denser in both cortex and medulla. Such immunolabeling is specific, since an unrelated antibody did not yield any significant fluorescence when applied on the thymus section from an infected mouse (D). Mice were infected with 10^5^ trypomastigote forms of the parasite (Colombian strain), and sacrificed 21 d later, at the peak of parasitemia. C, cortex; Cap, capsule; M, medulla; S, septum. Bar represents 100 μm for all photomicrographs. Pictures were kindly provided by Désio Aurélio Farias-de-Oliveira.

In addition to soluble moieties, the intrathymic production of ECM is altered in infectious diseases (Figure 4). By using immunohistochemistry, we found increased deposition of ECM components such as laminin, fibronectin, and type IV collagen in various human and experimental acute infectious diseases, including rabies, syphilis, measles, Chagas disease, and schistosomiasis [32]. One could argue that such an increased in the intrathymic contents of ECM molecules merely reflects the atrophy of the organ, with a densification of the ECM-containing network as a result of thymocyte loss. Although such mechanical response is likely to occur, at least in experimental Chagas disease it does not solely account for the ECM increase, since in vitro T. cruzi infection of cultured thymic epithelial cells, as well as TEC cultures derived from in vivo-infected animals, does result in a enhancement of ECM production [62]. Moreover, it is interesting to note that in thymuses from T. cruzi acutely infected mice, thymocytes exhibit an increase in membrane density of ECM receptors for fibronectin and laminin [63]. As detailed below, such features are likely related to alterations in the migratory patterns of thymocytes.

#### Intrathymic T cell migration in infectious diseases: Putative relationship with release of potentially autoreactive cells.

Changes in the patterns of peripheral T cell migration have been reported in infectious diseases, and can been demonstrated in Chagas disease [64–66]. Such changes are obviously necessary in T cell-dependent immune responses mounted against the infectious agent, although they can generate autoimmune events. Although relatively few data are available on migratory disturbances of T lymphocytes within the thymus, we obtained evidence that thymocyte migration is altered in experimental T. cruzi infection. As mentioned above, TNC complexes can be considered an in vitro model of thymocyte migration in the thymic epithelium. In mice experimentally infected with *T. cruzi,* we found a decrease in the number and size of TNCs [50,62], although thymocyte release from the remaining lymphoepithelial complexes was faster than from corresponding controls, an event likely due to the enhancement of ECM production. Similar results were observed when TNC complexes from normal animals were infected in vitro [62].

Considering that most intra-TNC thymocytes are immature CD4^+^CD8^+^ cells, one could raise the hypothesis that the migratory capacity of these cells is enhanced in murine Chagas disease. Accordingly, we found a significant increase in the relative and absolute numbers of CD4^+^CD8^+^ cells in peripheral lymphoid organs of T. cruzi-infected mice in both acute and chronic phases of the disease. These lymphocytes are actually T cells since they express variable amounts of CD3 and TCR on their membranes [63,64]. Moreover, the thymic dependence of this increase in peripheral CD4^+^CD8^+^ T lymphocytes was demonstrated by the fact that it could be prevented in animals that were thymectomized prior to infection [63].

**Figure 5:**
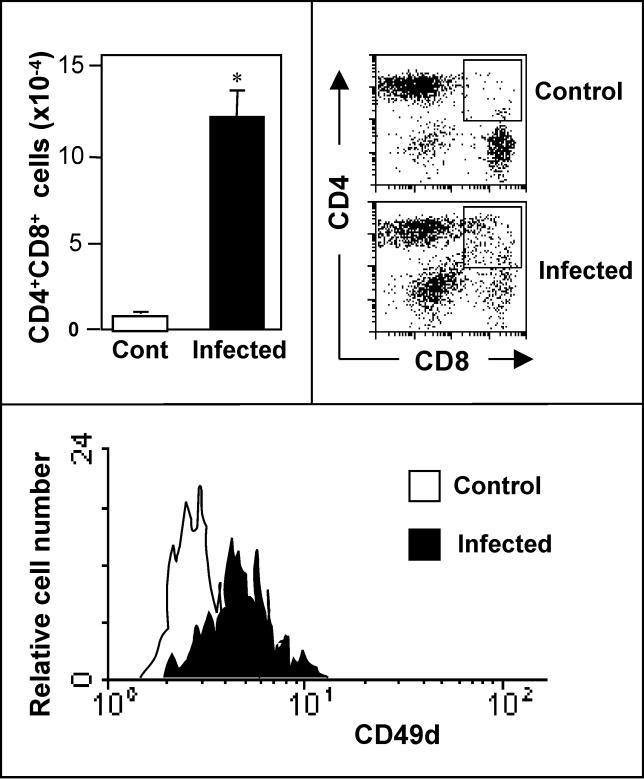
Appearance of Immature Thymus-Derived T Cells in Lymph Nodes of *T. cruzi-*Infected Mice The bar graph at the upper left shows the significant increase in the absolute numbers of CD4^+^CD8^+^ lymphocytes seen in subcutaneous lymph nodes of acutely infected mice. The flow cytometry dot plots to the right show the enhancement of these CD4^+^CD8^+^ cells in the infected animal and compared to the age-matched control. These immature double-positive T cells express higher amounts of the fibronectin receptor VLA-4, as ascertained by the cytofluorometric detection of the CD49d integrin subunit (bottom graph). Adapted from [43].

As seen in Figure 5, CD4^+^CD8^+^ peripheral T cells seen in experimental Chagas disease exhibited higher amounts of ECM receptors including the integrins VLA-4, VLA-5, and VLA-6 [65–69], indicating that an abnormal ECM-mediated interaction could be favoring the release of immature thymocytes from the organ.

More recently, we found that multiple driving forces likely favor export of CD4^+^CD8^+^ cells from the thymus of infected animals. Following acute T. cruzi infection, intrathymic CXCL12 contents increase and thymocytes from infected animals express higher levels of the corresponding receptor CXCR4. Accordingly, CXCL12 has a synergic effect with fibronectin in enhancing ex vivo migratory response of these cells [61].

A second aspect deserving comment is that some of the CD4^+^CD8^+^ cells seen in the periphery of infected animals appears to have bypassed intrathymic events of negative selection. In experiments performed on lymph nodes of acutely or chronically infected BALB/c mice, we found immature CD4^+^CD8^+^ cells as well as mature T cells bearing “forbidden” TCRs that should have been deleted in thymus, including those belonging to the Vβ5 and Vβ12 TCRβ families [64]. Conjointly, these data indicate that such CD4^+^CD8^+^ cells abnormally released from the thymus of T. cruzi infected have bypassed intrathymic negative selection, thus bearing a potential autoreactive phenotype that apparently differentiates in the periphery into CD4^+^ or CD8^+^ single-positive cells [64]. In this respect, it is noteworthy that CD4^+^ T cell-mediated autoreactivity against myocardial cells has been experimentally demonstrated in murine Chagas disease [68,69].

The findings discussed above illustrate the notion that the ability of the thymus to release lymphocytes into the periphery of the immune system is crucial for determining the role of this organ in the pathophysiology of infectious disease. This ability can be evaluated through the analysis of the so-called “recent thymic emigrants” (RTEs) [11]. In experimental animals, these cells can be tracked in the periphery, following intrathymic labeling of lymphocytes with fluorescein isothiocyanate. The FITC^+^ cells seen in peripheral lymphoid organs or in the blood are those recently exported from the thymus. Alternatively, RTEs can be evaluated by the presence of T cell excision circles (TRECs), circular DNA fragments derived from the rearrangement of TCR genes that remain within mature thymocytes and RTEs. This is the method mostly used for determining RTEs in humans.

In the case of experimental Chagas disease, the idea is plausible that changes in thymocyte migration are at the origin of the release of potentially autoreactive T cell clones. Nevertheless, this notion remains as a hypothesis, since the actual autoimmune nature of these cells has not been established. In a broader sense, it will be worthwhile to search for potentially autoreactive cells in other infectious diseases, to better understand the role of the thymus in these conditions.

Despite the abnormal release of immature thymocytes seen in acute T. cruzi infection, the overall rate of mature thymocyte export in infectious disease is likely lower than what is seen in normal conditions, due to the low absolute numbers of these cells in the thymus. Because of the degree of thymic atrophy seen in these acutely infected animals, tracing RTEs in the periphery of the immune system by intrathymic injection of fluorescein isothiocyanate was not technically feasible (unpublished data), and the analysis of TREC^+^ cells has not been done so far.

By contrast, TREC analysis has been performed in human and simian immunodeficiency virus infections, and in both cases the numbers of TREC^+^ T lymphocytes in the peripheral blood were lower than in uninfected individuals [18,70]. Importantly, specific highly active antiretroviral therapy (HAART) in AIDS patients promoted an increase in recent thymic emigrants, or TREC^+^ cells, in the blood [71]. Actually, this effect could be further enhanced by using HAART plus growth hormone treatment [72]. Patients considered poor responders to HAART exhibited minimal thymic tissue (as defined by computer tomography scanning) and had significantly fewer circulating TREC^+^CD4^+^ T cells than did the good HAART responders [73], indicating that poor CD4^+^ T cell replenishment in treated AIDS patients may in part reflect decreased thymic function. In addition, long-term survivors of pediatric HIV infection showed recovery of thymic volume and numbers of circulating TRECs to levels that reached values similar to uninfected age-matched individuals [74].

Modulation of thymocyte export in AIDS may be a direct effect of virus-derived proteins on T cells. It has been determined that the HIV nef regulatory protein alone is able to inhibit CXC12-induced migration of peripheral CD4^+^ T cells by interfering with the CXCR4 downstream signaling pathway [75].

A different scenario has been drawn for measles virus-infected children, in which an increased relative number of TREC^+^ cells were reported despite the existence of thymic atrophy [76]. Nevertheless, this conclusion should be made with caution, since in parallel with the slight (although statistically significant) relative increase of circulating RTEs, the authors also reported that the same children had severe lymphopenia that corresponded to a 50% decrease in total numbers of lymphocytes. Thus, in terms of absolute cell export from the thymus, a decrease (rather than an increase) in exit of thymocytes may occur following human measles virus infection.

#### Anti-thymus antibodies in infectious diseases.

In addition to the mechanisms discussed above, in terms of the changes seen in both lymphoid and microenvironmental compartments of the thymus with infectious diseases (that in some cases may be related to an abnormal release of potentially autoreactive cells), there is evidence that the thymus itself is a target of autoimmune events. For example, we found anti-TEC and anti-thymocyte antibodies in both acute and chronic phases of experimental and human Chagas disease [27,77]. In human syphilis, we observed immunoglobulin deposits in basement membrane of thymic lobules and increased numbers of B cells within the thymic lobules (unpublished data). Intrathymic deposits of immunoglobulins and complement, as well as plasma cells, were also reported in AIDS patients [27,78]. Importantly, circulating self-reactive antibodies from AIDS patients promoted massive thymocyte destruction when injected into normal mice [79]. Thus, it is possible that intrathymic antibody deposition may play a role in thymic functions under the context of a given infectious disease. This hypothesis obviously needs to be better evaluated, but represents an interesting field of investigation.

#### Intrathymic detection of infectious agents.

**Figure 6:**
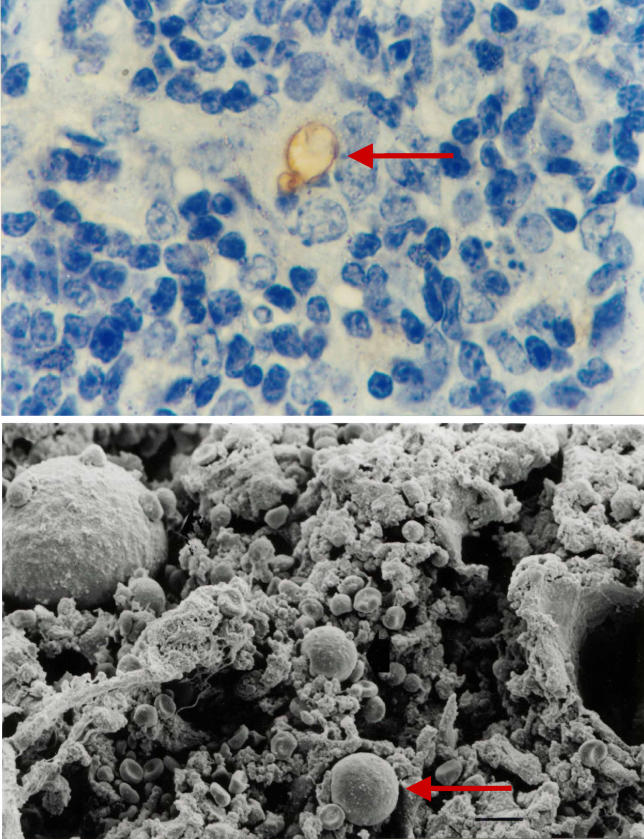
Intrathymic Presence of the Fungus P. brasiliensis Fungus particles (red arrows) are shown by immunohistochemistry (upper image) and scanning electron microscopy (lower image). Note that infective particles are encircled by microenvironmental cells bearing large nuclei. Pictures were kindly provided by Dr. Liana Verinaud.

The various aspects discussed above, concerning both the lymphoid and microenvironmental changes of the thymus in infectious diseases, lead to an obvious question: To what extent are these alterations due to a direct intrathymic effect of the given infectious agent? To answer this question a first approach is to define whether or not the infectious agent (or respective-derived moieties) can be detected intrathymically. Rather surprisingly, despite the existence of the so-called blood-thymus barrier, infectious agents have been detected within the organ, including viruses (HIV, SIV, lymphocoriomeningitis, etc.), protozoan parasites such as *T. cruzi,* and even fungi, as exemplified by Paracoccidioides brasiliensis in Figure 6.

**Figure 7:**
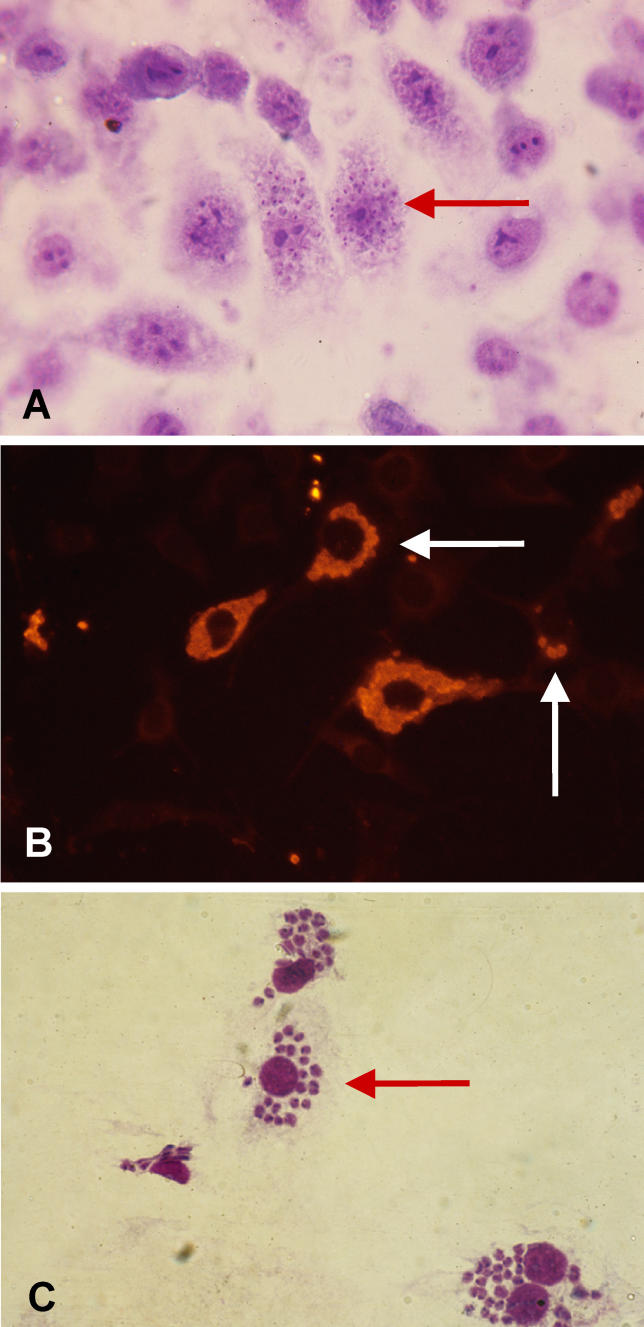
In Vitro Infection of Mouse Thymic Microenvironmental Cells by T. cruzi The presence of the amastigote forms of the parasite within cultured thymic epithelial cells was ascertained by Giemsa staining (A) and by immunohistochemistry (B). Infected thymic phagocytic cells are shown in (C). Arrows indicate intracellular amastigote clusters. Pictures were kindly provided by Désio Aurélio Farias de Oliveira.

We found T. cruzi parasites in both thymic macrophages and epithelial cells [53]. Accordingly, these microenvironmental components can be infected in vitro, as seen in Figure 7. In this case, intrathymic infection may play a direct role in generating thymic atrophy, since the parasite-derived trans-sialidase is likely at the origin of thymocyte death seen in acutely infected animals [50].

Measles virus can also infect the thymic epithelium, both in vivo and in vitro. Studies using human TEC cultures showed that the virus can enter the cells via a specific membrane protein termed the nucleoprotein receptor [80].

Choriomeningitis virus, as well as HIV and SIV, are able to infect thymic lymphocytes. Moreover, it is likely that HIV infects the thymic microenvironment, since a significant viral load was found in the microenvironmental compartment of the thymus in HIV-infected SCID-hu mice [55], a finding that was recently confirmed by the demonstration of infected thymic dendritic cells [81]. Whether or not the thymic epithelium is also infected by this virus is an issue that deserves further investigation.

Conceptually, the fact that some infectious agents enter the thymus and infect thymic cells raises the hypothesis of central tolerance for at least some infectious agent-derived antigens. This issue remains largely unexplored and represents a relevant field for further investigation.

Conversely, intrathymic manipulation also offers a potential way to enhance the ability of T cells to control infection. This will be hopefully be achieved through the use of so-called “thymic vaccination.” The concept is based on the fact that slightly altered peptides bearing lower affinity to the corresponding TCR than to the natural cognate ligand may induce positive selection of this molecule when injected intrathymically, leading to antigen-specific T cell export from the thymus [82,83]. Accordingly, in theory it should be possible to enhance the immune response against a given infection agent by increasing the numbers of positively selected thymocytes able to recognize a given molecule of the corresponding infectious agent.

#### Conclusions and perspectives.

The many aspects discussed above clearly illustrate that the thymus is a target organ in a variety of infectious diseases. The dynamic alterations in thymocyte subpopulations, including proliferation and death, the shaping of the T cell repertoire, and quantitative and qualitative migratory changes in thymocyte subpopulations, are likely related to events that take place later in the periphery of the immune system, thus influencing the pathophysiology of a given infection.

Although thymic atrophy is one common feature in a variety of infectious diseases, the mechanisms driving such thymocyte death apparently vary according to a given infection, and thus deserve detailed investigation.

Another important issue that needs to be further developed is the presence of a given infectious agent within the thymus versus the potential impact on the generation of the T cell repertoire, including tolerance to some peptides derived from the infectious organism.

In conclusion, more studies on the thymus in the context of infectious diseases are needed, to better understand the behavior of this organ in respect to thymocyte differentiation under the biological pressure of a given infection. These studies should address the generation of the T cell repertoire and potential autoimmune events (including cell death and cell expansion) and the export of cells toward the periphery. Such a systematic approach will certainly be helpful in improved design of specific immunotherapeutic interventions.

We should also consider attempts to enhance antigen-specific expansion of the thymus-derived repertoire through thymic vaccination with altered ligand peptides. Furthermore, modulators of thymocyte migration and proliferation should be considered in order to yield larger numbers of antigen-specific T cells exiting the thymus and colonizing T cell regions of peripheral lymphoid organs—procedures that should result in the enhancement of the corresponding immunological response.

Lastly, the surprising (yet consistent) data unraveling a functional second thymus in the adult mouse [84] places on the stage a whole additional set of studies that should be carried out in order to see, at least in the mouse model, if a neck-located thymus is equally sensitive to the various infections discussed in this review.

## Abbreviations

ECM: extracellular matrix
HAART: highly active antiretroviral therapy
IL: interleukin
MHC: major histocompatibility complex
RTE: recent thymic emigrant
SIV: simian immunodeficiency virus
TCR: T cell receptor
TEC: thymic epithelial cell
TNC: thymic nurse cell
TNF: tumor necrosis factor
TREC: T cell excision circle
